# An examination of daily CO_2_ emissions prediction through a comparative analysis of machine learning, deep learning, and statistical models

**DOI:** 10.1007/s11356-024-35764-8

**Published:** 2025-01-13

**Authors:** Adewole Adetoro Ajala, Oluwatosin Lawrence Adeoye, Olawale Moshood Salami, Ayoola Yusuf Jimoh

**Affiliations:** 1 Centre of Excellence for Data Science Artificial Intelligence & Modelling (DAIM), University of Hull, HU6 7RX Hull, United Kingdom; 2 Department of Geology and Mineral Science, Kwara State University, Malete, P.M.B. 1530, Ilorin, Kwara State Nigeria; 3 Research Division, Stalwart Ventures LLC, 5719 Cedonia Avenue, Suite F, Baltimore, MD 21206 USA

**Keywords:** Daily CO_2_ emissions, Prediction and forecast, Machine learning model, Deep learning model, Statistical model

## Abstract

Human-induced global warming, primarily attributed to the rise in atmospheric CO_2_, poses a substantial risk to the survival of humanity. While most research focuses on predicting annual CO_2_ emissions, which are crucial for setting long-term emission mitigation targets, the precise prediction of daily CO_2_ emissions is equally vital for setting short-term targets. This study examines the performance of 14 models in predicting daily CO_2_ emissions data from 1/1/2022 to 30/9/2023 across the top four polluting regions (China, India, the USA, and the EU27&UK). The 14 models used in the study include four statistical models (ARMA, ARIMA, SARMA, and SARIMA), three machine learning models (support vector machine (SVM), random forest (RF), and gradient boosting (GB)), and seven deep learning models (artificial neural network (ANN), recurrent neural network variations such as gated recurrent unit (GRU), long short-term memory (LSTM), bidirectional-LSTM (BILSTM), and three hybrid combinations of CNN-RNN). Performance evaluation employs four metrics (*R*^2^, MAE, RMSE, and MAPE). The results show that the machine learning (ML) and deep learning (DL) models, with higher *R*^2^ (0.714–0.932) and lower RMSE (0.480–0.247) values, respectively, outperformed the statistical model, which had *R*^2^ (− 0.060–0.719) and RMSE (1.695–0.537) values, in predicting daily CO_2_ emissions across all four regions. The performance of the ML and DL models was further enhanced by differencing, a technique that improves accuracy by ensuring stationarity and creating additional features and patterns from which the model can learn. Additionally, applying ensemble techniques such as bagging and voting improved the performance of the ML models by approximately 9.6%, whereas hybrid combinations of CNN-RNN enhanced the performance of the RNN models. In summary, the performance of both the ML and DL models was relatively similar. However, due to the high computational requirements associated with DL models, the recommended models for daily CO_2_ emission prediction are ML models using the ensemble technique of voting and bagging. This model can assist in accurately forecasting daily emissions, aiding authorities in setting targets for CO_2_ emission reduction.

## Introduction

Human-induced global warming presents a significant and urgent threat with widespread implications for the environment and society (Kong et al. 2022). This increase in the Earth’s average temperature, resulting from the emission of greenhouse gases (nitrous oxide-N_2_O, carbon dioxide-CO_2_, methane-CH_4_, ozone-O_3_, and chlorofluorocarbon-CFC), gives rise to severe consequences, including elevated sea levels, extreme weather events, reduced crop yields, water scarcity, and disturbances to ecosystems (Legg 2021). These outcomes, in turn, pose substantial risks to human well-being. Among the various greenhouse gases released, CO_2_ contributes to approximately 81% of total emissions (Amarpuri et al. 2019; Rehman et al. 2021).

The 2021 global carbon budget revealed that 33% of total CO_2_ emissions over the past 70 years occurred after the turn of the millennium (Huang et al. 2023). This substantial release of CO_2_ is a major contributor to the issue of global warming (Fakana 2020). The correlation between CO_2_ and global warming is rooted in this gas’s ability to trap heat in the Earth’s atmosphere, resulting in a greenhouse effect that elevates the Earth’s temperature. The increase in atmospheric CO_2_ levels is attributed primarily to the combustion of fossil fuels, and China, the USA, the EU27&UK, and India account for most of the global emissions (Crippa et al. 2021).

To address the challenge of global warming, the Paris accord, established under the United Nations Framework Convention on Climate Change (UNFCCC), was adopted in 2015. The agreement aims to constrain global warming to either 2 or 1.5 °C by implementing policies that reduce emissions, ultimately achieving net-zero carbon by 2050 (Azevedo et al. 2021). Additionally, in 2021, approximately 200 countries signed the Glasgow Climate Pact (COP26), which aims to encourage governments to “accelerate the development, implementation, and dissemination of technologies, and the adoption of policies to shift towards a low-carbon emission energy system” (Lennan and Morgera 2022). However, the development of effective policies for setting carbon emission mitigation targets requires the attainment of highly accurate prediction models, thus presenting a new area of challenge. Consequently, there is growing significance in research dedicated to the prediction of CO_2_ emissions.

**Fig. 1:**
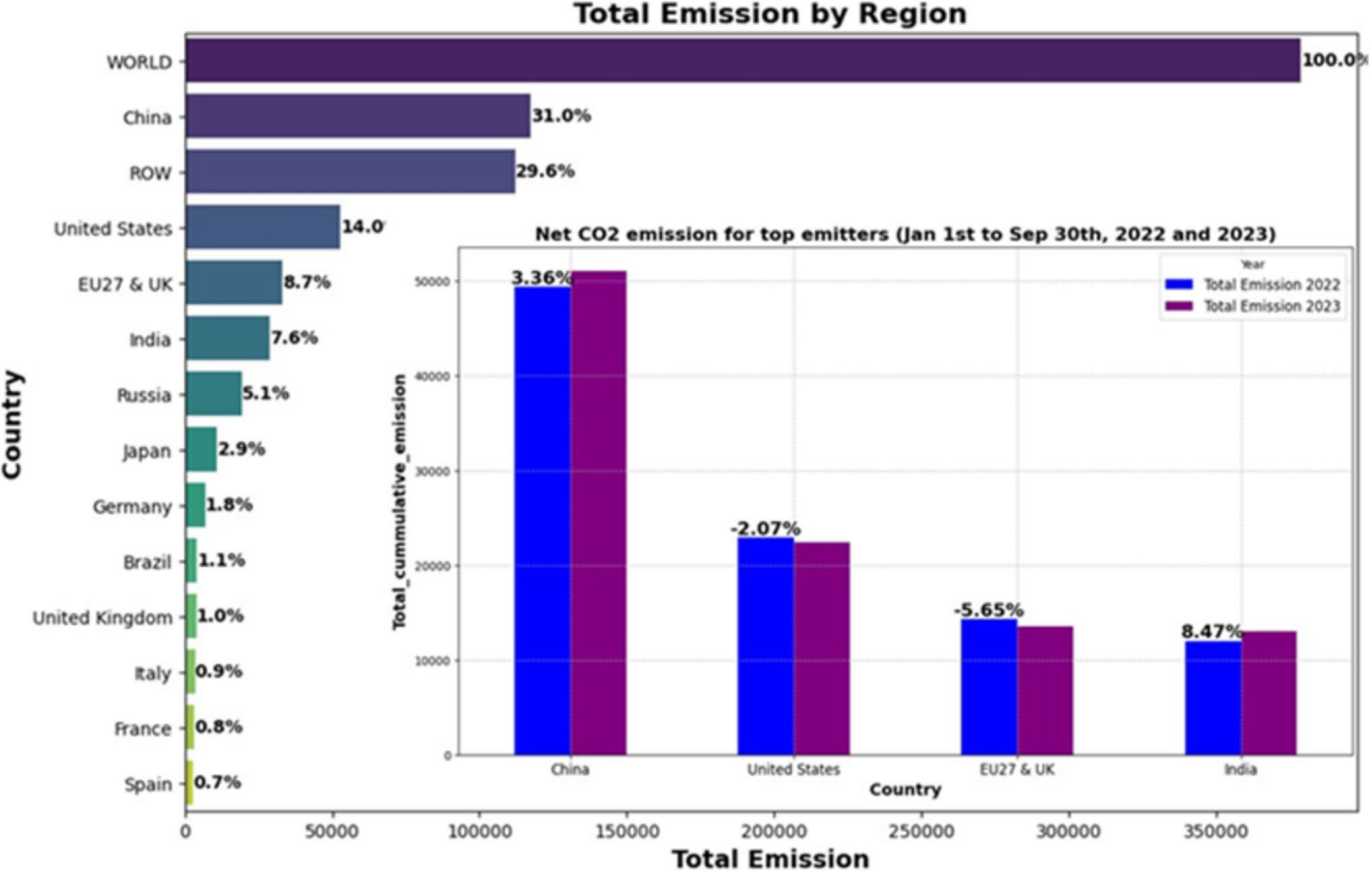
Global CO_2_ emissions between 1/1/2022 to 9/30/2023

Currently, most of the research is focused on predicting annual CO_2_ emissions. While this approach is essential for establishing long-term emission mitigation targets, it has limitations. These limitations arise from its small sample size and its inability to capture daily and monthly fluctuations, which are critical for setting actionable emission reduction policies. In contrast, adopting daily real-time carbon emission prediction allows monitoring of dynamic trends and fluctuations in CO_2_ emissions. This approach proves valuable for establishing short-term emission targets, allowing time for effective countermeasures and better control over carbon emissions. Hence, the exploration of daily emission prediction is highly important. Furthermore, an initial exploratory analysis of global CO_2_ emissions, as shown in Fig. 1, reveals that more than 61% of total global CO_2_ emissions originate from four regions (China, India, the USA, and the EU27&UK), with China and India characterised by a net emission increase. Therefore, understanding these regions’ current and future daily carbon dioxide emission levels is crucial.

This study investigates the performance of four statistical models (ARMA, ARIMA, SARMA, and SARIMA), three machine learning models (SVM-support vector machine, RF-random forest, and GB-gradient boosting), and seven deep learning models (ANN-artificial neural network, and RNN-recurrent neural network variation of GRU-gated recurrent unit, LSTM-long short-term memory, BILSTM-bidirectional-LSTM, and three Hybrid combinations of CNN-RNN) in predicting daily CO_2_ emission across four regions (China, India, USA, and EU27&UK). The performance of these models is evaluated via four metrics, and a comparative analysis is conducted to identify the best model for daily CO_2_ emission prediction. The dataset used for this study comprises daily CO_2_ emissions data from 1/1/2022 to 9/30/2023 (638 data points), reflecting post-COVID-19 normal economic activities. This systematic approach ensures that the selected model delivers accurate predictions and demonstrates robustness and reliability. The possible innovations and contributions of this paper are as follows:

- Bridging the Gap in Daily CO_2_ emissions prediction: A key contribution of this research is its focus on addressing the critical gap in daily carbon dioxide emissions prediction, which is essential for policymakers in the timely adjustment of actionable short-term emission reduction strategies. Unlike most existing studies that focus on annual carbon dioxide emissions, this study concentrates on the often-overlooked aspect of daily emissions. Doing so fills a significant void in the field and provides valuable insights that can directly inform and enhance policy decision-making.
- Comprehensive model evaluation: This study conducts a thorough evaluation of various prediction models, including statistical, machine learning, and deep learning approaches. The analysis identifies the most effective models for daily CO_2_ emission forecasting and provides insights into their strengths and limitations. This evaluation offers valuable guidance for future research in selecting the most appropriate predictive models for daily CO_2_ emission forecasting.
- Expansion of geographical scope: Unlike previous studies, which focused primarily on daily emissions in China, this study extends the analysis to include four major polluting regions: China, India, the USA, and the EU27 & UK. By evaluating the performance of prediction models across these diverse regions, the study provides a more comprehensive perspective on the applicability and generalizability of the models, thus enhancing their relevance to a variety of datasets.
- Incorporation of data transformation techniques: This study applied data transformation methods, such as differencing, to improve the accuracy of daily CO_2_ emission predictions, particularly in machine learning models that do not rely on the assumption of stationarity. This approach not only improves current predictive capabilities but also offers a foundation for further advancements in the accuracy of carbon emission forecasting in future research.

This study presents new insights backed by empirical evidence, along with practical recommendations aimed at enhancing the accuracy of daily carbon dioxide emission predictions. As a comprehensive analysis, it serves as a valuable resource for researchers, facilitating the development of effective emission reduction strategies and supporting informed environmental policymaking.

The remainder of the paper is organized as follows: the “Literature review” section comprehensively reviews previous work on CO_2_ emission forecasting and related studies, establishing the foundation for the present study. The “Methodology” section introduces the data sources, proposed models, and evaluation criteria for assessing the prediction models. This section details the data selection and collection process, outlines the methodologies applied in the prediction models, and describes the metrics used to evaluate model performance. The “Results and discussion” section then presents a detailed evaluation and comparison of each prediction model’s performance, examining the results to emphasise their strengths, limitations, and overall efficacy. This section also explores the implications of the findings and their relevance to the research objectives. Finally, the “Conclusion”, “Limitations and Future Work” sections summarise the key findings, discusses policy implications, and outlines limitations and potential avenues for future research.

### Literature review

Many scientific studies have aimed to increase the precision of CO_2_ emission prediction. These studies can be broadly categorised into two groups on the basis of the input data: multivariate and univariate methods. The multivariate approach incorporates multiple factors influencing CO_2_ emissions as model inputs. However, this method has a major disadvantage: obtaining complete data for all influencing factors is often challenging in practical applications. As a result, missing data are frequently extrapolated, which may lead to inaccurate predictions or the generation of an erroneous model (Song et al. 2023). Additionally, assessing the individual contribution of each influencing factor is complex. In multivariate models, interactions between variables are often intricate and interdependent, making it difficult to isolate and quantify the impact of specific factors. Wang et al. (2019) reported that multicollinearity, where predictors are highly correlated, can distort model coefficient interpretations, complicating the identification of individual factor impacts. Prakash and Singh (2024) addressed the missing data issue by employing linear interpolation and mean method techniques to replace missing values in their study on CO_2_ emissions from coal power plants. While these techniques help address data gaps, they also introduce uncertainty, potentially affecting model accuracy.

In contrast, the univariate approach relies on readily available historical data, reducing uncertainties in model assumptions and predictions (Ziel and Weron 2018). This method avoids the complexities and challenges associated with missing data in multivariate models, making it preferable when high-quality, comprehensive datasets are unavailable or difficult to obtain. Several recent studies (Giannelos et al. 2023; Kour 2023) have demonstrated that the univariate approach can yield robust predictions with simpler input structures, mitigating risks associated with incomplete or interpolated data. Existing studies on univariate CO_2_ emission prediction can be further divided based on the predictive models employed: traditional statistical models and artificial intelligence (AI) models (Mason et al. 2018). Traditional statistical time series models, such as Holt-Winters, ARIMA, and SARIMAX, are commonly applied for short-term CO_2_ emission prediction. However, these statistical methods have two significant limitations: (1) they are effective for stationary time series data but struggle with nonlinear cases, and (2) they are not ideal for predicting large sample sizes (Ren et al. 2016; Kong et al. 2022). Despite these limitations, several studies have utilised these models for CO_2_ emission prediction. For example, Kour (2023) and Kumari and Singh (2022) effectively applied statistical models to forecast annual CO_2_ emissions in South Africa (using ARIMA) and India (using SARIMAX), respectively. However, Li and Zhang (2023) reported that these models performed poorly when applied to daily CO_2_ emission data.

Given that daily CO_2_ emission data often display nonlinear fluctuations, the linearity assumption inherent in statistical models may lack validity (Peng et al. 2020). Consequently, there has been a shift toward the use of traditional machine learning (ML) and deep learning (DL) models, which are better suited for large datasets and nonlinear data, as highlighted by Zhou et al. (2021). Numerous studies have since focused on predicting CO_2_ emissions using traditional ML and DL techniques. These AI models include support vector machines (Adegboye et al. 2024), random forests (Zhang et al. 2023b), gradient boosting regressors (Romeiko et al. 2020), artificial neural networks (ANNs) (Ağbulut 2022), and recurrent neural networks (RNNs), such as LSTM (Huang et al. 2019) and BiLSTM (Aamir et al. 2022), along with hybrid combinations of convolutional neural networks (CNNs) and RNNs (Amarpuri et al. 2019; Faruque et al. 2022). These models have been applied across various sectors, such as emissions from buildings (Giannelos et al. 2023), CO_2_ emissions from transportation (Ağbulut 2022), emissions from coal power plants (Prakash and Singh 2024), CO_2_ emissions in the industrial sector (Song et al. 2023), CO_2_ emissions from diverse fuel sources (Jeniffer et al. 2023), and energy consumption (Adegboye et al. 2024). A summary of recent studies using the univariate approach for CO_2_ emissions prediction is provided in Table 1.

**Table 1:** Studies on univariate CO_2_ emissions prediction in the past 5 years

Reference literature	Technique	Dataset	Best model	Metrics
Nyoni and Bonga (2019)	ARIMA	1960–2017 (Annual)	ARIMA (2,2,0)	RMSE, MAE & MAPE
Kour (2023)	ARIMA	1960–2016 (Annual)	ARIMA (4,2,3)	RMSE, MAE & MAPE
Giannelos et al. (2023)	ARIMA, Linear Regression, Shallow & Deep Neural Network (NN)	1971–2014 (Annual)	Deep NN	MAPE
Kumari and Singh (2022)	ARIMA, SARIMAX, Holt-Winters, LR, RF, SVM, ANN and LSTM	1980–2019 (Annual)	LSTM	R^2^, MAE, MSE, RMSE, MAPE, MSLE, ME, & MedAE
Geevaretnam et al. (2022)	ARIMA, RF, SVM, ANN	1991–2020 (Annual)	SVM	MAE, RMSE & MAPE
Amarpuri et al. (2019)	CNN-LSTM, Exponential Smoothing	1960–2017 (Annual)	CNN-LSTM	*R*^2^, MAE, RMSE & MAPE
Huang et al. (2019)	PCA + LSTM, BPNN, GPR	1990–2016 (Annual)	PCA + LSTM	MAPE
Song et al. (2023)	EMD, Elman Neural Network, Univariate Polynomial Regression (TVF-EMD-ENN-UPR)	01/01/2019–31/05/2022 (daily)	TVF-EMD-ENN-UPR	R^2^, RMSE & MAPE
Kong et al. (2022)	ICEEMDAN + Hybrid Prediction Model	01/01/2019–18/06/2021 (daily)	ICEEMDAN -ISSA-ELM	R^2^, RMSE & MAPE
Qiao et al. (2020)	SVM Optimised with Lion Swarm Optimizer and Genetic Algorithm	1965–2017 (annual)	SVM	MAE
Li and Zhang (2023)	GM, ARIMA, SARIMAX, ANN, RF, LSTM	01/01/2020–30/09/2022 (daily)	LSTM	*R*^2^, MAE, MSE, RMSE & MAPE,

As shown in Table 1, most prior studies have focused primarily on annual CO_2_ emissions. While annual predictions are essential for setting long-term mitigation targets, they have limitations, including small sample sizes and an inability to capture daily or monthly fluctuations, which are crucial for actionable short-term policies. In contrast, daily real-time CO_2_ predictions allow monitoring of dynamic trends and fluctuations, which is invaluable for setting short-term targets and implementing timely interventions. Consequently, daily emissions prediction represents a critical area for further exploration. Additionally, studies by Magazzino and Mele (2022) and Magazzino et al. (2023) illustrate the potential of combining neural networks with time series decomposition to capture emissions’ dynamic behaviour and improve accuracy. Although these studies applied multivariate approaches, they provide insights that complement our strategy of integrating data transformation techniques, such as differencing, to enhance the performance of ML and DL models.

Building on advancements in CO_2_ emission prediction, this study introduces several key innovations to address existing gaps. Our research provides a comparative evaluation of 14 models, highlighting variations in performance and identifying the best-performing model. This study broadens the geographical scope by assessing models across China, India, the USA, and the EU27&UK, enhancing the generalizability and applicability of predictive methods. Additionally, by applying data transformation techniques, such as differencing, we improve the accuracy of daily CO_2_ emission predictions, especially for the ML and DL models that benefit from stabilised data. By analysing recent daily data from January 2022 to September 2023, reflecting post-COVID-19 economic activity, this study offers empirical evidence and novel insights that meaningfully contribute to the body of literature on short-term CO_2_ emission forecasting.

## Methodology

### Dataset

The dataset consists of 638 daily real-time CO_2_ emission data points from January 1, 2022, to September 30, 2023, sourced from the carbon monitoring project (https://carbonmonitor.org) (Liu et al. 2022). This period was specifically chosen to reflect normal economic activities following the global recovery from the COVID-19 pandemic. The CO_2_ emissions, measured in MtCO_2_/day (million tons of CO_2_ per day), were calculated as the total contributions from six sectors (domestic and international aviation, ground transportation, power, industrial, and residential) for China, India, the USA, and EU27&UK.

**Fig. 2:**
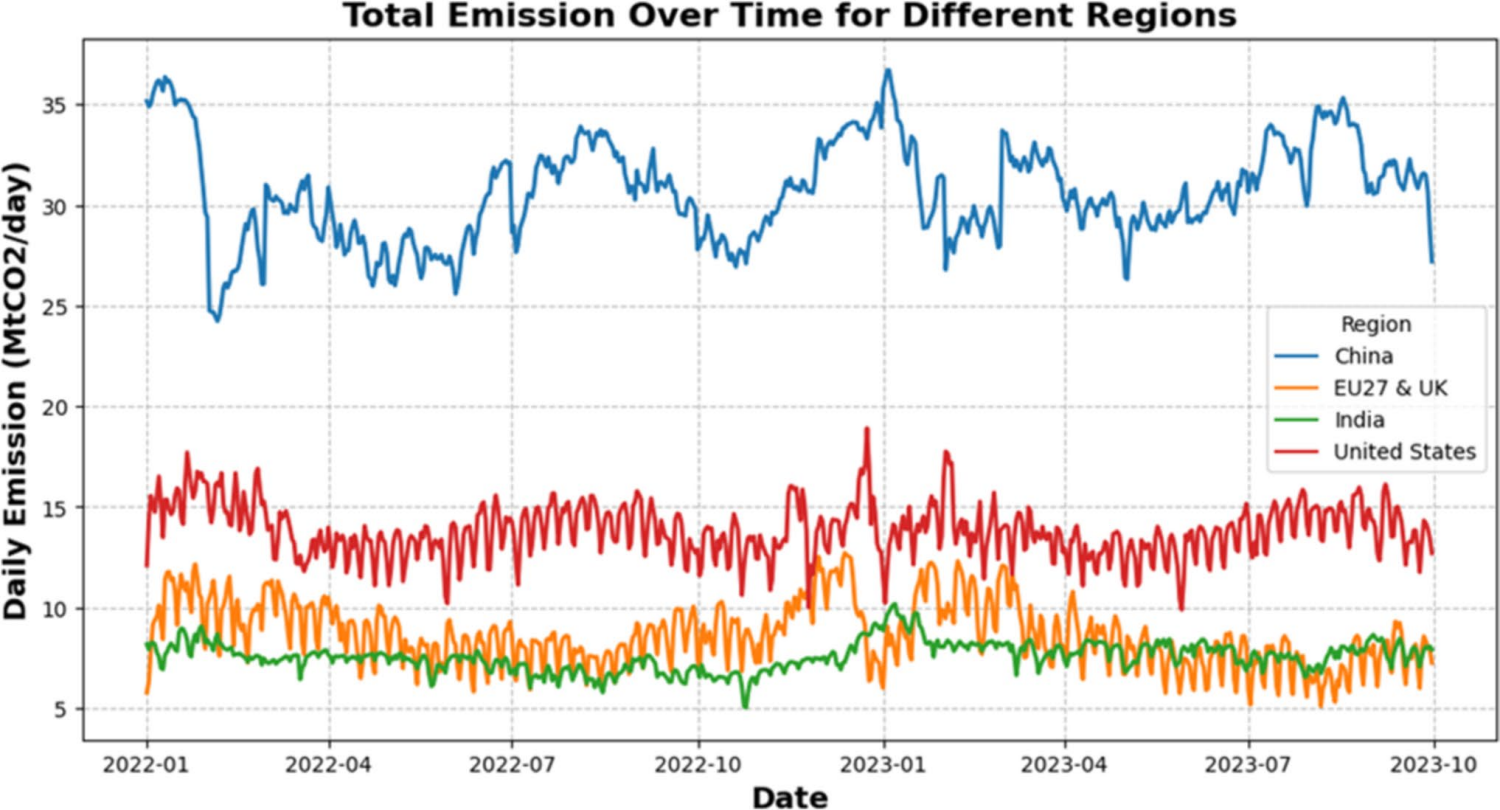
Daily CO_2_ emissions for the four regions

The data for the four regions exhibit high nonlinearity and non-stationarity, as shown in Fig. 2. The descriptive statistics in Table 2 show that China has the highest variability in daily CO_2_ emissions, followed by the USA and the EU27&UK, whereas India has the least variability, as indicated by its lower standard deviation. The median values are close to the mean across regions, suggesting relatively symmetrical distributions. However, kurtosis and skewness reveal that China and the EU27&UK have lighter tails, indicating fewer extreme emission values. Despite India’s lower overall variability, its higher kurtosis and skewness suggest occasional spikes in emissions, similar to those in the USA. China had the highest emissions, with a mean of 31 MtCO_2_/day, approximately double that of the USA (14 MtCO_2_/day), the second-highest emitter. The coefficient of variation (CV) was calculated to assess the relative variability in daily CO_2_ emissions. China, with the highest absolute emissions, shows stable emissions (CV = 8.03%), whereas the EU27&UK exhibit greater volatility (CV = 18.29%), indicating more significant fluctuations and clarifying the consistency of emissions patterns across regions.

**Table 2:** Descriptive statistics of daily CO_2_ (MtCO_2_/day) emissions

Country	Count	Mean	Min	Max	Median	Kurtosis	Skewness	StandardDeviation	CV/%
China	638	30.65	24.23	36.65	30.60	− 0.44	0.01	2.46	8.03
USA	638	13.83	9.90	18.91	13.77	0.45	0.15	1.30	9.40
EU27&UK	638	8.64	5.10	12.72	8.46	− 0.37	0.35	1.58	18.29
India	638	7.57	5.03	10.18	7.57	0.94	0.26	0.72	9.51

Seasonal variations in CO_2_ emissions can be attributed to several factors, including energy demand driven by weather. As shown in Fig. 2, peaks in CO_2_ emissions around December and January across all datasets likely reflect increased energy consumption for heating during colder months, whereas warmer periods show higher emissions from air conditioning. Additionally, variations in economic activity, such as shifts in industrial production and transportation patterns, may contribute to temporary spikes or drops in emissions. Thus, the seasonality in CO_2_ emissions arises from a combination of weather-related energy demands, economic cycles, and other temporal factors.

### Data preprocessing

No imputation for missing data was necessary, as the dataset contained no missing values. The main data preprocessing steps are as follows:

#### Stationarity check

The Augmented Dickey-Fuller (ADF) test was utilised to assess the stationarity of the datasets, which is vital for ensuring a consistent mean and standard deviation over time. Stationarity is critical for accurate predictions, particularly in statistical models that rely on this assumption. Although machine learning (ML) and deep learning (DL) models can process non-stationary data, they generally perform better with stationary inputs (Zhou et al. 2021). The ADF test results include both a test statistic and a *p*-value. The test statistic is compared against critical values to determine whether the null hypothesis (that the data are non-stationary) can be rejected. A more negative test statistic provides stronger evidence against the null hypothesis, indicating that the series is stationary. The *p*-value, derived from this test statistic, quantifies the strength of this evidence, with a *p*-value below 0.05 suggesting stationarity (Ajewole et al. 2020). As shown in Table 3, the China dataset was stationary without any transformation, as indicated by a highly negative test statistic of − 3.9787 and a *p*-value of 0.0015. In contrast, the USA, the EU27&UK, and India datasets were non-stationary in their original form, as indicated by their less negative test statistics and *p*-values greater than 0.05. After applying first-order differencing, these datasets achieved stationarity, which was reflected in more negative test statistics and significantly lower *p*-values, well below the 0.05 threshold for rejecting the null hypothesis and assuming stationarity.

**Table 3:** ADF test results on the original and transformed data

Dataset	Data transformation	Test statistics	*P*-value	Conclusion
China	No transformation	− 3.9787	0.0015	Stationary
	No transformation	− 2.7903	0.0597	Non-stationary
USA	Differencing	− 2.9936	0.0350	Stationary
	No transformation	− 2.7380	0.0677	Non-stationary
EU27&UK	Differencing	− 5.5470	1.6524 * 10^^−6^	Stationary
	No transformation	− 2.5694	0.0995	Non-stationary
India	Differencing	− 5.8816	3.0687* 10^^−7^	Stationary

*P*-value < 0.05 = Stationary

#### Windowing technique

Windowing is a crucial process in transforming time series data into a supervised learning dataset, which is essential for machine learning (ML) and deep learning (DL) models. This technique involves partitioning the time series data into smaller, uniformly sized windows, often called lags. Each window contains a sequence of successive data points that capture the temporal dependencies within the dataset (Moroney 2019). By sliding a fixed-size window across the time series, we can generate input features and corresponding target labels for model training. This sequential iteration allows the model to learn from past values to predict future outcomes, effectively enabling time series forecasting. In this study, a window size of 7 was used. Specifically:

- 1 $$\text{Input Features }= [{\text{X}}_{1}, {\text{X}}_{2}, \dots , {\text{X}}_{7}], [{\text{X}}_{2}, {\text{X}}_{3}, \dots , {\text{X}}_{8}], \dots , [{\text{X}}_{n-7}, {\text{X}}_{n-6}, \dots , {\text{X}}_{n}]$$
  Here, X_*i*_ represents the data points at each time step, and the window size of 7 captures seven consecutive time points for generating the input features.
- 2 $$\text{Labels }= [{\text{X}}_{8}], [{\text{X}}_{9}],\dots ,[{\text{X}}_{n+1}]$$

Each input feature vector consists of seven consecutive time points, such as [X_1_, X_2_, …, X_7_]. The corresponding label is the time point immediately following this window, for example, X_8_. This process is repeated across the entire time series, producing input features and labels for all possible windows in the dataset. For the final window, which includes the last data point X_n_, the label is X_n+1_, which follows the last window. This windowing technique helps the model focus on short-term dependencies while allowing it to generalise across different time intervals within the dataset. It is widely used in various applications, including stock market prediction, weather forecasting, and other domains where temporal patterns are critical (Patel et al. 2015; Faruque et al. 2022).

### Models

The study explored four statistical models, three machine learning models, and seven deep learning models to predict and forecast daily CO_2_ emissions. The detailed components of each model are explained in the subsequent sections.

#### Statistical models

The ARIMA and ARMA models are widely used statistical models that combine autoregression (AR) and moving average (MA) techniques. In ARIMA, “I” signifies integration, indicating the differencing order required to make the time series stationary (Kumari and Singh 2022). The parameters for AR, I, and MA are represented as (p, d, q), where p represents past values for predictions, d is the order of differencing ensuring stationarity, and q is the lag number of prediction errors used to enhance the current timestamp (Kour 2023). An ARIMA model is derived when these parameters are greater than 0. When d equals 0, it corresponds to the ARMA model. The ARIMA model can be expressed mathematically as follows.

3 $${\varphi }_{p}\left(B\right){\left(1-B\right)}^{d}{y}_{t}={\theta }_{q}\left(B\right){\varepsilon }_{t}$$

where ${\varphi }_{p}$ = autoregressive coefficient; B = backshift operator; d = order of differencing; ${y}_{t}$ = original time series data; ${\theta }_{q}$ = moving average; and ${\varepsilon }_{t}$ = white noise or error term (Cho et al. 1995). The main advantage of ARIMA and ARMA models is their simplicity and effectiveness in capturing linear patterns. However, these models have certain limitations. One drawback is that they require the original or differenced time series data to be stationary. Additionally, they may not perform well with nonlinear data, as they are more attuned to linear patterns (Li and Zhang 2023).

Introducing seasonality into the modelling process can enhance the model’s predictive performance when dealing with seasonal data (Kumari and Singh 2022). Unlike ARMA and ARIMA, SARIMA incorporates additional parameters specifically tailored for capturing seasonal patterns. These parameters include the seasonal autoregressive parameter (AR) P, seasonal differencing D, and seasonal moving average (MA) Q, denoted as P, D, Q, and s, respectively. Collectively, the SARIMA model integrates both seasonal and nonseasonal components, resulting in a total of p, d, q, P, D, Q, and s parameters, represented as SARIMA (p, d, q) × (P, D, Q, s). The mathematical expression of the SARIMA model is as follows:

4 $${\Phi }_{p}{({B}^{s})\varphi }_{p}\left(B\right){\left(1-{B}^{s}\right)}^{D}{\left(1-B\right)}^{d}{y}_{t}={\Theta }_{Q}{({B}^{s})\theta }_{q}\left(B\right){\varepsilon }_{t}$$

where ${B}^{s}$ = the seasonal reverse potential; ${\Phi }_{p}$ = the seasonal AR coefficient; ${\Theta }_{Q}$ = the seasonal MA coefficient; and ${\left(1-{B}^{s}\right)}^{D}$ = the seasonal differencing of the order D (Lee and Han 2020).

The model selection process for these statistical models typically begins with ensuring stationarity in the time series data by estimating the necessary differencing orders (d and D). Once stationarity is achieved, the appropriate model parameters (p, d, q for ARIMA/ARMA; P, D, Q, s for SARIMA) are identified via visual tools such as PACF and ACF plots (Sen et al. 2016). These parameters are then fine-tuned through hyperparameter tuning to obtain the optimal configuration that minimises the Akaike Information Criterion (AIC) and Bayesian Information Criterion (BIC), ensuring the most effective balance between model complexity and goodness of fit. After fitting the model to the training data, residual analysis is performed to detect any systematic patterns or errors in the residuals, ensuring that the model’s errors are random and unbiased. Model validation is then conducted by comparing the model’s predictions against actual test data and assessing performance metrics such as the R-squared value. Once validated, the model is used to make predictions on new, unseen data, providing insights into future trends or patterns in the time series.

#### Machine learning models

This study employed support vector regressor (SVR), ensemble methods such as random forest (RF), and gradient boost regressors (GB) because of their successful application in CO_2_ emission prediction, as reported by several researchers (Kumari and Singh 2022; Ağbulut 2022; Romeiko et al. 2020).

- Support Vector Regressor (SVR)

SVR is based on statistical learning theory and works by mapping the input data into a higher-dimensional feature space using kernel functions (Vapnik 1998). In this transformed space, it finds the optimal hyperplane that separates the data while maximising the margin of tolerance or error, effectively balancing complexity and accuracy. This hyperplane acts as a predictive model, estimating the output for new data. The basic equation for the SVM can be expressed as:

5 $$f\left(x\right)=\sum\limits_{i=i}^{n}{\alpha }_{i }K\left({x}_{i},x\right)+b$$

where ${\alpha }_{i}$ represents the model parameters (Lagrange multipliers), $K\left({x}_{i},x\right)$ represents the kernel function that transforms the data into higher dimensions, and b is the bias term (Mohammadi et al. 2015).

The most commonly used kernel functions are the linear, polynomial, and radial basis functions (RBF). The choice of the kernel function plays a critical role in capturing linear and nonlinear relationships in daily CO_2_ emission patterns. Linear and RBF kernels are used in this study because they are relatively simple to implement and involve tuning fewer parameters than more complex kernels while providing effective predictive performance (Zhang et al. 2021). The linear kernel works well with linearly separable data, whereas the RBF kernel captures nonlinear relationships efficiently.

The performance of the SVR is optimised by tuning three key parameters: the kernel function, epsilon (ϵ), and the regularisation parameter *C.* The epsilon value defines a margin of tolerance within which predictions are considered accurate, which helps to ensure that the model generalises well to unseen data. The *C* parameter controls the trade-off between minimising errors on the training data and maintaining a smooth model; higher values of *C* lead to fewer errors but may risk overfitting, whereas lower values encourage a simpler model that may tolerate some errors (Kleynhans et al. 2017).

For CO_2_ emission forecasting, SVR offers several advantages. It can model complex and nonlinear patterns in the data, making it robust for handling real-world time series data that may exhibit irregularities and fluctuations. By adjusting the kernel, epsilon, and *C* parameters, SVR can effectively learn from historical emission data and make reliable future predictions, providing insights into long-term CO_2_ emission trends (Wang et al. 2023).

b) **Ensemble model**

In addition to SVR, ensemble methods such as random forest regressor and gradient boosting regressor also provide significant advantages for CO_2_ emission prediction (Wang et al. 2023; Zhang et al. 2023b). Ensemble learning leverages the combined strength of multiple machine learning models trained on the same dataset. Consequently, compared with individual machine learning models, ensemble models can increase forecast accuracy.

##### Random forest (RF)

The concept of random forests was introduced by Breiman (2001). Random forest is an ensemble learning method that uses decision trees as its basic units and combines them with the bagging technique to enhance performance. During training, the bootstrap resampling technique is applied to randomly select k samples from the original dataset, constructing k weak decision trees (Breiman 2001). Each decision tree grows independently, without constraints or pruning, and remains uncorrelated with the others. The model’s final output is determined by averaging the predictions (for regression) or through majority voting (for classification) (Tang and Zhang 2013). Averaging predictions across multiple models serves to reduce variance and enhance the stability of the trees’ predictive performance. Random forests excel in capturing complex nonlinear relationships between inputs and outputs, making them particularly effective for regression tasks (Fan 2017). The fundamental equation for ensemble prediction is as follows: for any given sample X with P sub-models, each sub-model will generate its own prediction value. Let ${Y}_{k}$ represent the predicted value of the k^th^ sub-model; the overall model ${Y}_{E}$ will produce the final result by averaging these predictions (Yoon 2021).

6 $${Y}_{E}=\frac{1}{P}\sum_{k=1}^{P}{Y}_{k}$$

A major issue with the random forest model is overfitting. This occurs when a model fits the training data almost perfectly (performs well on the training set) but struggles to generalise to new, unseen data. Fully developed trees can lead to overfitting by capturing noise in addition to the underlying patterns in the training data. To address this issue, several adjustments can be made to the random forest model. Pruning the trees by reducing the maximum depth or limiting the number of nodes can help prevent overfitting. This study employed hyperparameter tuning to enhance the model’s performance. The key parameters considered included the maximum depth of the trees, the number of estimators, and the minimum number of samples required to split an internal node, as presented in Table 4.

##### Gradient boosting regressor (GB)

In contrast, the gradient-boosting regressor builds trees sequentially, with each subsequent tree aiming to rectify the errors of its predecessors (Friedman 2001). The model begins by constructing the first regression tree and then iteratively builds subsequent trees, splitting the data into smaller groups at each step. After each tree is built, the model evaluates the errors and trains the next tree to address those mistakes. This process continues until either the specified number of trees is reached or no further improvement in fit is possible. To prevent overfitting, gradient boosting uses a learning rate to scale the contribution of each new tree (Yoon 2021). A lower learning rate improves the model's ability to generalise, reducing the likelihood of overfitting by controlling how much each tree contributes to the final model. The key parameters tuned to improve the gradient boosting model's performance include the trees' maximum depth, the number of estimators, and the learning rate.

Overall, the grid search method was used to select the optimal parameters needed to enhance the performance of the RF, GB, and SVR models, as outlined in Table 4. Additionally, ensemble methods such as voting, bagging, and stacking were applied to further improve the models’ accuracy.

**Table 4:** Tested parameters for the machine learning models

SVR	RF	GB
C: {0.1,1,10}	n-estimators: {50,100,200}	n-estimators: {50,100,200}
Kernel: {linear, rbf}	max-depth: {range (1,6)}	Learning rate: {0.01, 0.1, 0.2}
Degree: {2,3,4}	Min-samples-split: {2, 5,10}	max-depth: {2,3,4}

#### Deep learning models

- Artificial neural networks (ANN)

**Fig. 3:**
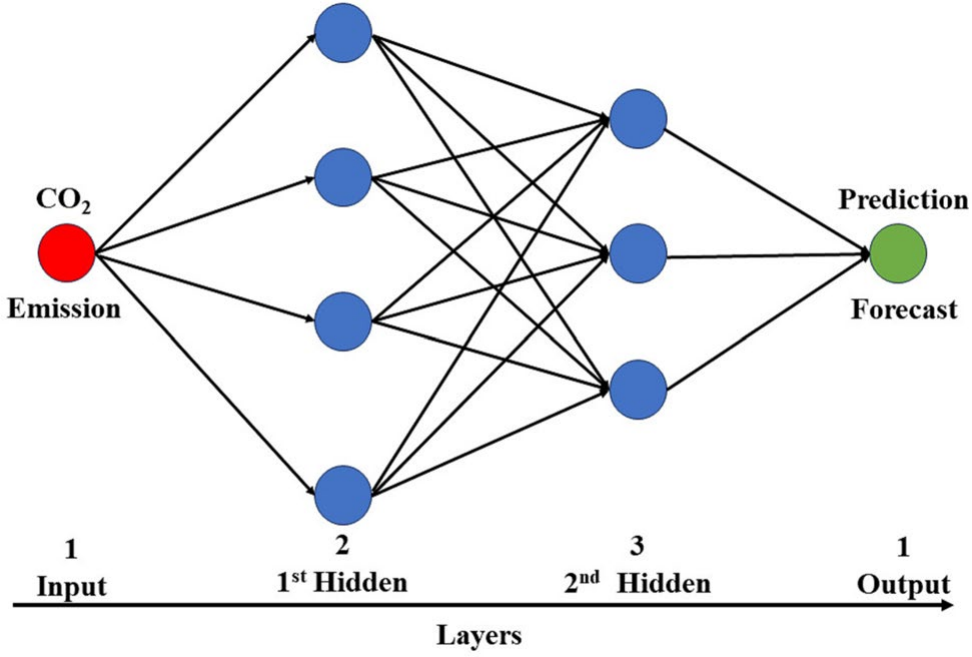
Schematic representation of the ANN model (modified after Ağbulut 2022)

Artificial neural networks are well-suited for capturing the complexities of nonlinear data, and their application in modelling CO_2_ emission data has been demonstrated by Ahmed et al. (2023). The strength of ANNs is their capacity for self-learning, which makes them particularly useful for managing nonlinear datasets such as daily emission data (Tümer and Akkuş 2018). A typical ANN consists of three layers, namely, the input-hidden-output layers, as shown in Fig. 3 (Ağbulut 2022). The hidden layer functions as the processing layer, enabling the model to learn patterns within the data. This layer consists of neurons (fundamental processing units) that receive input data from the input layer. The hidden layers adapt and learn based on the selected learning method for each processing layer, subsequently transmitting the acquired knowledge to the final layer, known as the output layer (Haider et al. 2022). The model parameters used in this research are shown in Table 5, and the mathematical expression for an ANN is presented as follows (Guo et al. 2021):

7 $${A}_{n}=\sum_{j=1}^{n}({w}_{j} .{I}_{j})+b$$

Here, *n* represents the number of inputs, ${w}_{j}$, and *b* correspond to the weight and bias, respectively, and ${I}_{j}$ represents the input and ${A}_{n}$ denotes the output of the ANN.

(b) Recurrent neural network (RNN)

Traditional convolutional neural networks (CNNs) are not designed to handle temporal dynamics, as they lack mechanisms for processing and propagating information across time steps. In contrast, recurrent neural networks (RNNs) are specifically designed to incorporate information from previous time steps into their current outputs (Ding et al. 2022). For daily CO_2_ emission prediction, recognising the time-dependent nature of the data is crucial, as it reveals a correlation between daily emission values over time. RNNs are particularly suitable for time-series modelling because of their loops in processing units, which facilitate the handling of sequential data. However, simple RNNs often struggle with capturing long-term dependencies, leading to issues such as vanishing and exploding gradient problems during training (Haider et al. 2022). This limitation can be mitigated by employing advanced RNN variants, such as gated recurrent units (GRUs), Long short-term memory (LSTM) networks, and bidirectional-LSTM (BILSTM) networks.

**Fig. 4:**
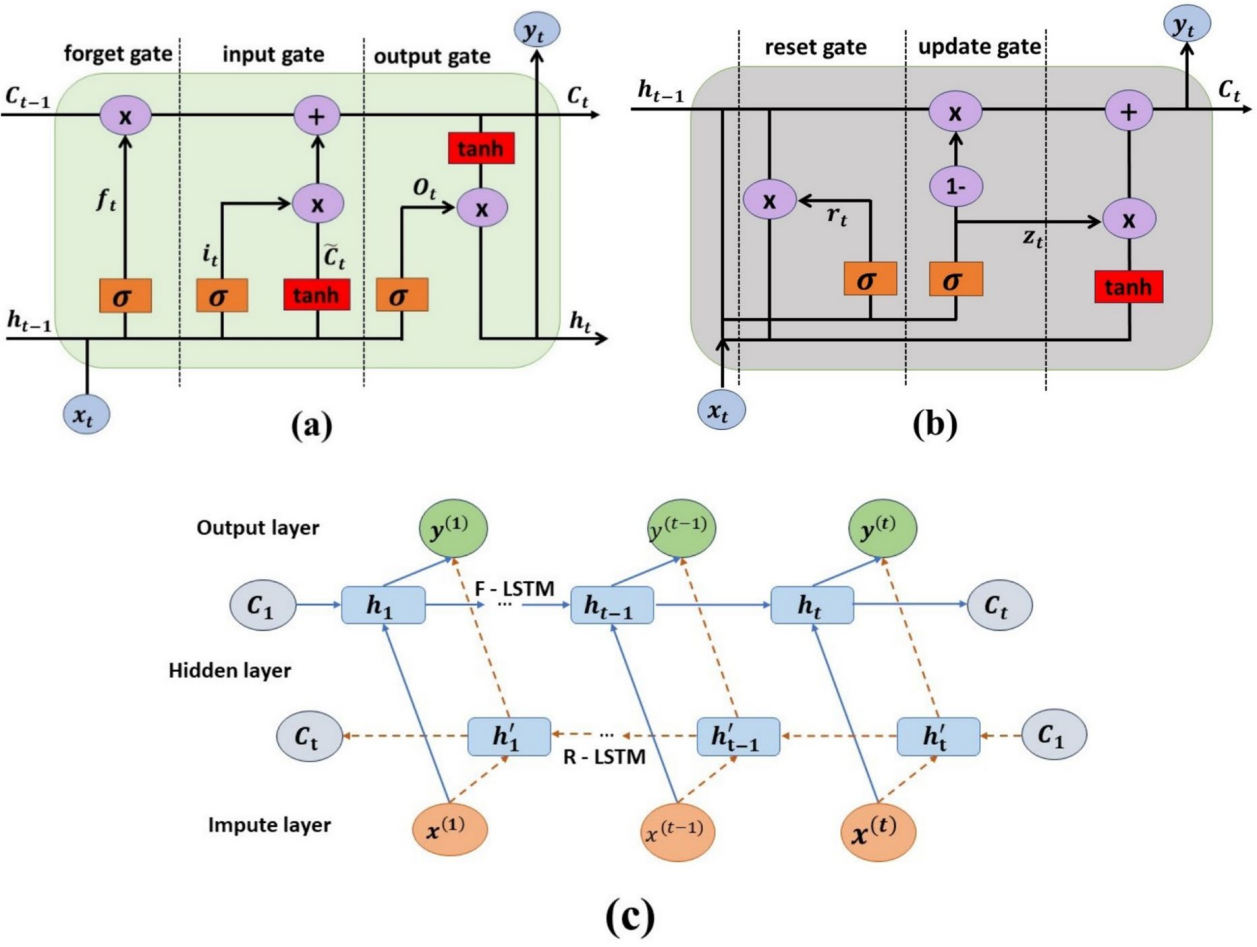
Schematic representation of the basin unit structure of the (**a**) LSTM and (**b**) GRU (**c**) BILSTM (modified after Ahem et al. 2023; Ding et al. 2022)

LSTM consists of a memory cell denoted by C_t_ and three gates: an input gate, a forget gate, and an output, which help retain and discard information as needed, effectively capturing long-term relationships (Hochreiter and Schmidhuber 1997). The architectural structure of the LSTM is presented in Fig. 4a, where the input, output, and forget gates are typically represented by ${i}_{t}$, ${O}_{t}$, and ${f}_{t}$ respectively. The input gate receives daily CO_2_ emission data, which the forget gate processes by merging with past hidden states. The forget gate determines which information from prior time steps should be retained or discarded. The concatenated input is passed through a nonlinear function, which stochastically updates values based on the current data. The output gate generates the predicted CO_2_ emissions, whereas a sigmoid layer, tanh layer, and pointwise operations such as summation and multiplication assist in the system's computations. The computing equation for an LSTM unit is presented as follows (Ding et al. 2022):

- Input:
  8 $${g}_{t}=\text{tanh}\left({W}_{ig}{x}_{t}+{b}_{ig}+{W}_{hc}{h}_{t-1}+{b}_{hg}\right)$$
- The Gate Status:
  9 $$input\;gate\left({i}_{t}\right)=\upsigma \left({W}_{ij}{x}_{t}+{b}_{ii}+{W}_{hi}{h}_{t-1}+{b}_{hi}\right)$$
  10 $$forget\;gate({f}_{t})=\upsigma \left({W}_{if}{x}_{t}+{b}_{if}+{W}_{hf}{h}_{t-1}+{b}_{hf}\right)$$
  11 $$Output\;gate\left({O}_{t}\right)=\upsigma \left({W}_{io}{x}_{t}+{b}_{io}+{W}_{ho}{h}_{t-1}+{b}_{ho}\right)$$
- Memory Cell:
  12 $${C}_{t}={f}_{t}{\times C}_{t-1}+{i}_{t}\times {g}_{t}$$
- The output:
  13 $${h}_{t}={O}_{t}+\text{tanh}({C}_{t})$$
  where *W* and* b* represent the weight and bias matrices, respectively. ${h}_{t-1}$ represents the hidden state (unknown vectors) from the previous time step. ${x}_{t}$ denotes the input at the current time step. These components collectively manage the flow of information within the LSTM, enabling it to capture relationships between various data points during prediction and retain critical information. As a result, LSTMs often produce more accurate results when handling time-series data predictions.

The GRU, a simpler variant of the RNN, is similar to the LSTM but uses two gates: the reset gate (combining the forget and input gates) and the update gate, which are typically denoted by ${r}_{t}$ and ${z}_{t}$, respectively, as shown in Fig. 4b (Hochreiter and Schmidhuber 1997). Reducing the number of gates contributes to GRUs’ faster training times and lower computational complexity. The key equations in the GRU are as follows (Ding et al. 2022):

- Update gate:
  14 $${z}_{t}=\upsigma \left({W}_{z}{x}_{t}+{R}_{z}{h}_{t-1}+{b}_{z}\right)$$
  The update gate provides adaptive regulation of information passing through the hidden unit, determining how much of the previous hidden state ${h}_{t-1}$ should be retained and combined with the incoming data for information updates.
- Reset gate:
  15 $${r}_{t}=\upsigma \left({W}_{r}{x}_{t}+{R}_{r}{h}_{t-1}+{b}_{r}\right)$$
  The reset gate controls how much of the past information should be reset or discarded.
- Candidate hidden state:
  16 $${h}_{t}{\prime}=\text{tanh}\left({W}_{u}{x}_{t}+{R}_{u}{({r}_{t}\times h}_{t-1})+{b}_{u}\right)$$
  The candidate’s hidden state ${h}_{t}{\prime}$ is computed via the reset gate-modulated previous hidden state and the current input ${x}_{t}$
- Final hidden state:
  17 $${h}_{t}= {z}_{t} \times {h}_{t}{\prime}+\left(1- {z}_{t}\right)\times {h}_{t-1}$$

The final hidden state ${h}_{t}$ is a combination of the candidate’s hidden state ${h}_{t}{\prime}$ and the previous hidden state ${h}_{t-1}$, weighted by the update gate ${z}_{t}$.

Compared with LSTM networks, which have three gates (input, forget, and output) and a separate memory cell, the simpler structure of a GRU, with fewer gates, results in lower computational complexity. This simplicity allows GRUs to train faster while still maintaining effective performance in capturing temporal dependencies, which can be beneficial for daily CO_2_ emission forecasting.

Compared with the traditional unidirectional LSTM, the BILSTM developed by Schmidhuber (2015) consists of two LSTMs with outputs in opposite directions, capturing information from both past and future time steps (seasonality), thereby improving the model's dependence and enhancing the model's overall forecasting accuracy. For BILSTM, the hidden layer ${h}_{t}$ consists of both forward ${{h}_{t}}^{\to }$ and backward ${{h}_{{t}{\prime}}}^{\leftarrow }$ LSTM units, which are expressed as follows:

18 $${h}_{t}={{h}_{t}}^{\to }\oplus {{h}_{{t}{\prime}}}^{\leftarrow }$$

where $\oplus$ denotes the element-wise summation of the forward and backward output components, and the BILSTM network structure is illustrated in Fig. 4c. Numerous researchers have used these three RNN variations for time series forecasting (Zhang et al. 2023a; Li and Zhang 2023; Huang et al. 2019).

Additionally, we explored a hybrid CNN-RNN model by incorporating a single convolutional layer into each RNN architecture. This integration allows the model to extract both spatial and temporal features simultaneously. The convolutional and pooling layers extract spatial features, whereas the RNN layers capture the time series information. This approach has shown potential for improving the forecast accuracy of time series models, as reported by numerous researchers (Faruque et al. 2022; Amarpuri et al. 2019).

**Fig. 5:**
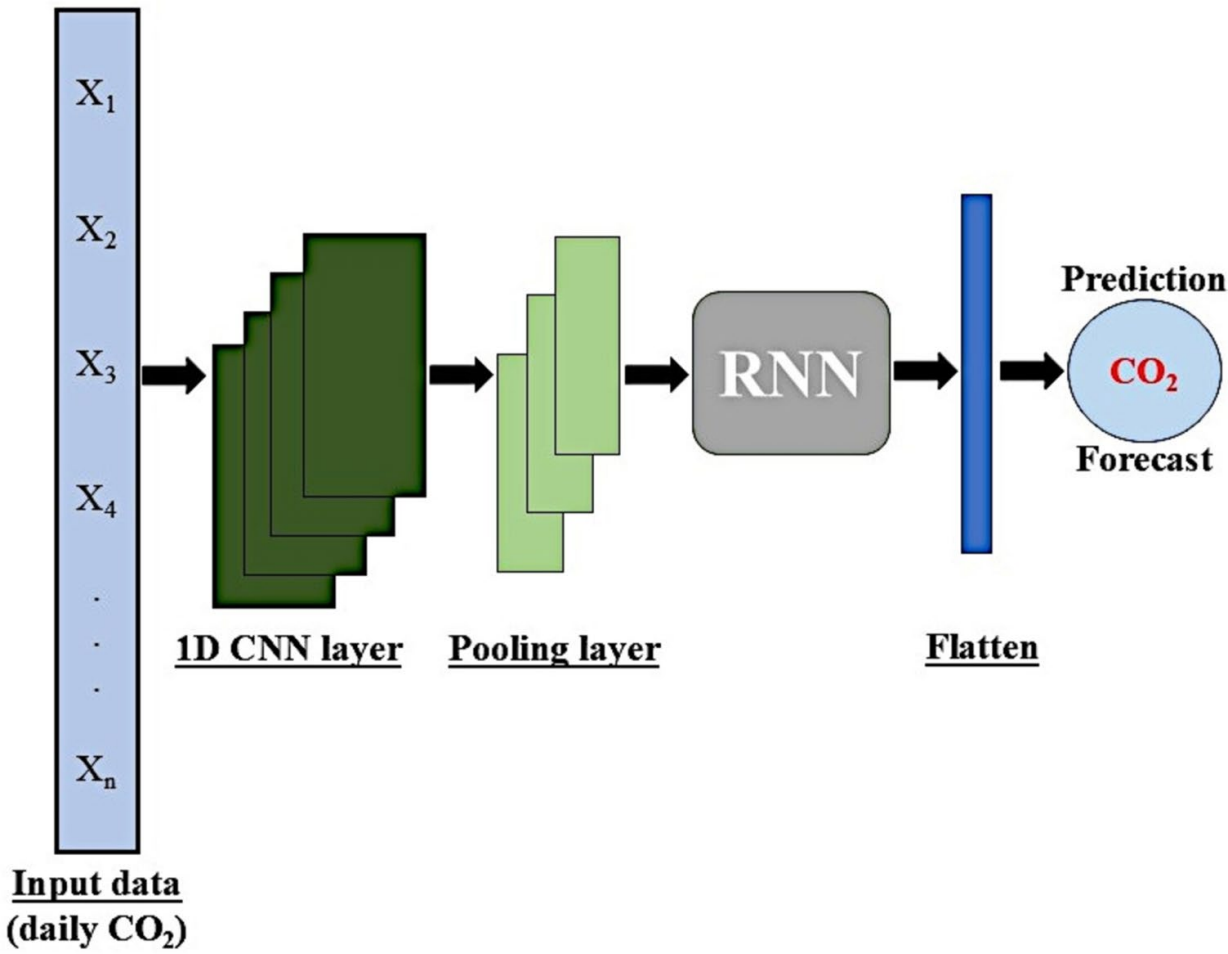
Architecture of the hybrid CNN-RNN model (modified after Faruque et al. 2022)

In the hybrid model, the input daily CO_2_ emission data are first fed into the CNN layer, where a convolution operation is applied to extract the characteristics of the complex, nonlinear interactions in the input data. The new feature matrix generated after the convolution operation is then passed to the pooling layer, which reduces the dimensionality of the feature map and helps prevent overfitting. This feature map is used as the input for the RNN layer. Finally, a fully connected layer is placed at the end to make the prediction, as shown in Fig. 5 below. The hybrid model uses a ReLU activation function for the input layers, whereas the output layer uses a linear activation function. The parameters of the deep learning models used in this study are listed in Table 5.

**Table 5:** Model parameters of the deep learning models

Parameters	ANN	GRU	LSTM	BILSTM	CNN-RNN
Kernal size	Na	Na	Na	Na	varied
Filter size	Na	Na	Na	Na	varied
Conv1D	Na	Na	Na	Na	1
Drop out	0.2	0.2	Varied	0.2	Varied
Number layer	3	3	3	3	4
Learning rate	0.001	0.001	0.001	0.001	varied
Optimizer	Adam	Adam	Adam	Adam	Adam
Batch size	32	32	32	32	32
No of Epochs	200	200	200	200	200
Error monitored	MSE	MSE	MSE	MSE	MSE

*Na*, not applicable

### Research workflow

**Fig. 6:**
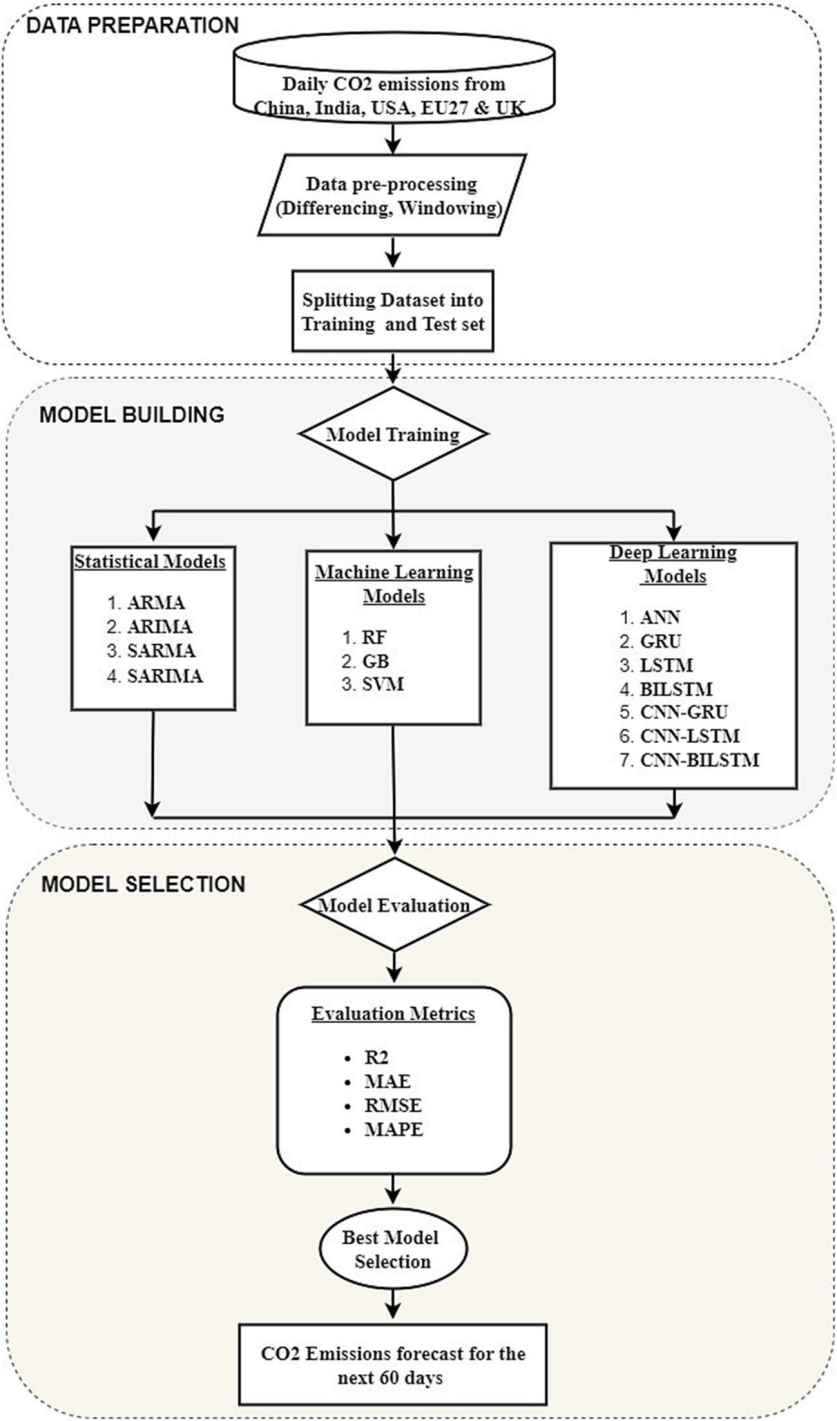
Research workflow

The research workflow is divided into three key phases: data preparation, model building, and model selection, as illustrated in Fig. 6.

1. Data preparation:

Daily univariate CO_2_ emission data for China, India, the USA, and the EU27&UK were collected. To make the data suitable for time series models, differencing was applied to ensure stationarity, which is essential for models such as ARIMA and SARIMA, which require stationary input data. Windowing techniques were employed to prepare the data for the ML and DL models, ensuring that temporal dependencies were captured. The dataset was split into training (80%) and testing (20%) sets to ensure robust model evaluation. The entire data preparation process was carried out via Python’s Panda library.

(2) Model building:

The conceptual design of this study involves comparing various models to determine which best fits the CO_2_ emission data across different regions. The choice of models was informed by their theoretical foundation and empirical success in previous CO_2_ emission forecasting studies (Shabani et al. 2021; Kumari and Singh 2022; Faruque et al. 2022). Specifically:

- Statistical models: ARMA, ARIMA, SARMA, and SARIMA were selected on the basis of their success in capturing linear trends and seasonal patterns in stationary time series data. Studies such as those of Kour (2023) and Kumari and Singh (2022) have demonstrated the efficacy of these models in CO_2_ emission forecasting.
- The machine learning models SVM, random forest (RF), and gradient boosting (GB) were chosen for their flexibility in handling nonlinear relationships and high-dimensional data, as demonstrated by Adegboye et al. (2024), Zhang et al. (2023b), and Romeiko et al. (2020). These models do not rely on stationarity assumptions and can capture interactions between variables that traditional statistical models might overlook.
- The deep learning models ANN, GRU, LSTM, and BILSTM were used to model long-term dependencies in the data. These models have been shown in studies such as those of Shabani et al. (2021), Huang et al. (2019), and Aamir et al. (2022) to be effective at capturing nonlinear, non-stationary patterns in CO_2_ emission datasets. Hybrid models that combines CNN and RNN components have also been explored to enhance the ability to capture complex temporal relationships, as Faruque et al. (2022) demonstrated. All modelling and analyses were performed using Python libraries such as scikit-learn (for the ML models), stats-models (for the statistical models), and TensorFlow/Keras (for the DL models). Hyperparameter tuning was conducted via a grid search to identify optimal model configurations.

(3) Model selection:

Model selection was based on a comprehensive evaluation using metrics such as the RMSE, MAE, MAPE, and *R*^2^, as recommended by Li and Zhang (2023). These metrics provide insights into both the scale and direction of prediction errors, offering a well-rounded assessment of model performance. Model comparison and visualisation were performed using Python’s matplotlib library. The final model selection was driven by both empirical performance and theoretical suitability, with the selected model used to forecast CO_2_ emissions for 60 days across the four major regions. This approach balances traditional statistical theory with modern ML/DL techniques to ensure that the most appropriate model is chosen for accurate daily CO_2_ emission forecasting.

### Performance evaluation

Model performance was evaluated via metrics such as the coefficient of determination (*R*^2^) and error metrics such as the root mean square error (RMSE), mean absolute error (MAE), and mean absolute percentage error (MAPE). Smaller values of the error metrics and a higher *R*^2^ value indicate better model performance. Table 6 provides a concise overview of the various evaluation metrics employed in this study.

**Table 6:** Summary of the evaluation metrics used in this study

Metrics	Equation	Description
RMSE	$=\sqrt{\sum\limits_{i=1}^{n}\frac{{\left({x}_{i}-{y}_{i}\right)}^{2}}{n}}$	It is the root of the MSE and has the same unit as the target variable, making it more interpretable. The closer the result is to zero, the better the performance of the model (Ağbulut et al. 2021)
MAE	$= \frac{\sum\nolimits_{i=1}^{n}\left({x}_{i}-{y}_{i}\right)}{n}$	It represents the sum of the absolute deviation of the prediction from the actual value, and a smaller value is desirable (Kumari and Singh 2022)
MAPE	$=\frac{100\%}{n}\sum\limits_{i=1}^{n}\left|\frac{{y}_{i}-{x}_{i}}{{y}_{i}}\right|$	It indicates the model's average prediction accuracy, with a smaller value being desirable (Song et al. 2023)
*R*^2^	$=1- \frac{{\sum }_{i=1}^{n}{\left({x}_{i}-{y}_{i}\right)}^{2}}{{\sum }_{i=1}^{n}{\left({y}_{i}-{x}_{i}\right)}^{2}}$	It varies from 0 to 1, denoting the level of correspondence between the predicted and actual values (Song et al. 2023). The more the value approaches 1, the better the performance of the model
	${y}_{i} , {x}_{i}=$	*Actual and predicted values*

## Results and discussion

### Results

#### Statistical models

On the basis of the performance metrics presented in Table 7, it is evident that the ARMA model is the best statistical model, exhibiting lower MAE, RMSE, and MAPE values, along with a higher *R*^2^ value across regions. However, the incorporation of differencing using ARIMA and SARIMA models resulted in a decrease in the overall model performance. For example, the USA dataset’s *R*^2^ decreased from 0.412 to 0.205 (ARIMA) and 0.321 (SARIMA). Similar observations occurred across all the other regions. This observation aligns with the findings of Kumari and Singh (2022), who emphasise that while differencing aids in achieving stationarity by removing trends and seasonality, it may also lead to a loss of valuable information, resulting in inaccurate predictions.

**Table 7:**
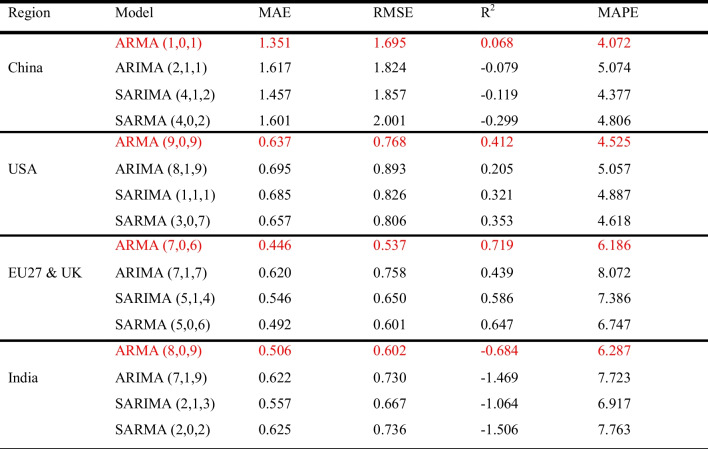
Statistical models performance evaluation

*The red mark indicates the best models*

The poor performance of ARIMA and SARIMA models in datasets such as those from China and India, as reflected by negative *R*^2^ values, underscores the limitations of these models in capturing the high variability and nonlinear patterns present in CO_2_ emission data. While differencing helps achieve stationarity, it may inadvertently remove significant information, particularly in highly volatile datasets such as those from China. Li and Zhang (2023) similarly highlighted that traditional models such as ARIMA and SARIMA struggle with nonlinear, volatile datasets.

**Fig. 7:**
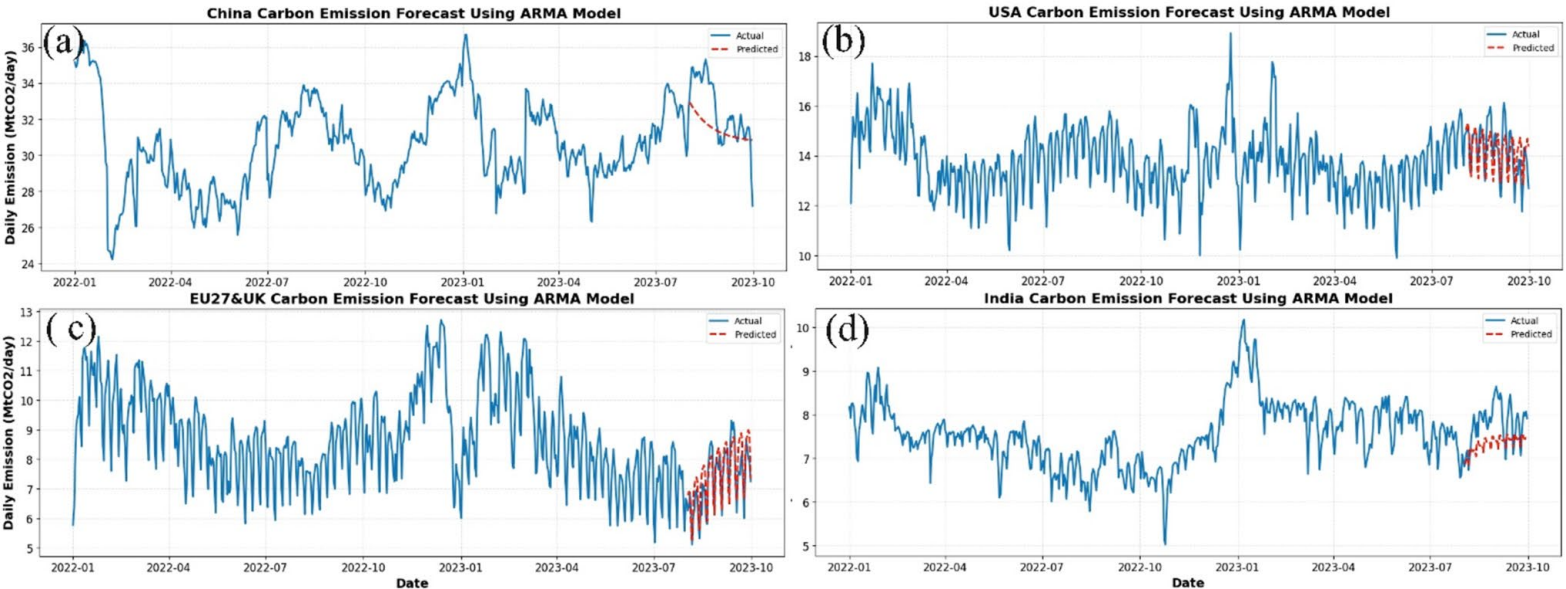
Plots of actual and predicted emissions for (**a**) China, (**b**) USA, (**c**) EU27&UK, and (**d**) India using the best statistical model

In summary, while ARMA performed moderately well in regions such as the USA and the EU27&UK, with *R*^2^ values of 0.412 and 0.719 respectively, its overall performance was suboptimal for the China and India datasets, where the *R*^2^ values were mostly negative (*R*^2^ ≤ 0.068), indicating that the predicted values significantly differ from the actual values, as illustrated in Fig. 7. These models’ limitations in handling nonlinearity and volatility make them less suitable for forecasting daily CO_2_ emissions.

#### Machine learning models

As demonstrated in Table 8, the performance of the ML models significantly improved with differencing compared with that of the statistical models. For example, differencing increased the *R*^2^ values of the RF model from 0.899 to 0.918, 0.585 to 0.804, 0.644 to 0.852, and 0.663 to 0.720 for China, the USA, the EU27&UK, and India datasets, respectively. A similar improvement trend was observed for all the other evaluation metrics (MAE, RMSE, and MAPE) across the four regions. While ML models such as RF, SVM, and GB do not inherently require stationarity, they can be sensitive to non-stationary data, leading to the model capturing spurious relationships and yielding inaccurate predictions (Hyndman 2018). By applying differencing to stabilise the time series mean, the models can better assume a constant mean, thereby improving their predictive accuracy.

**Table 8:**
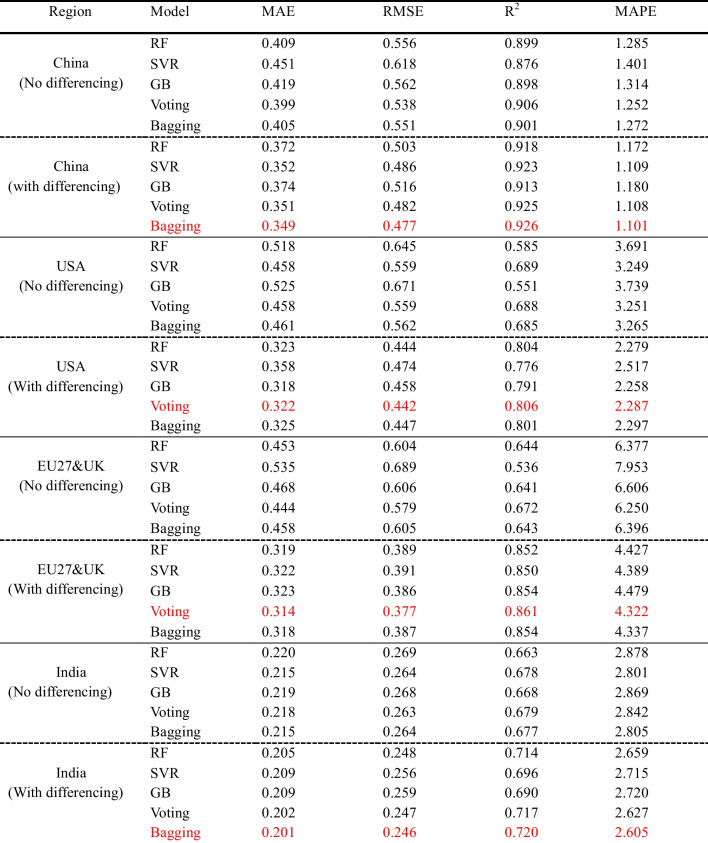
Machine learning models performance evaluation

*The red mark indicates the best models*

Seasonal patterns in daily CO_2_ emissions significantly influence regional variations in model performance. In regions such as China and the USA, emissions peak during December and January due to increased energy consumption for heating during the winter months. These seasonal fluctuations are more pronounced in China because of its larger industrial base and reliance on coal-powered energy sources (Zhang et al. 2019). Conversely, the EU27&UK region also displays clear seasonal peaks, although these tend to be less pronounced than those in China. This can be attributed to the more diversified energy sources and stringent emission regulations within the EU, which have helped curb emissions (European Environment Agency 2021). In contrast, India shows a comparatively less pronounced seasonal pattern, which may be due to lower variability in energy demand throughout the year, owing to its tropical climate and lower per capita energy consumption (Vishwanathan and Garg 2020).

The seasonal patterns of daily CO_2_ emissions introduce significant nonlinearity and non-stationarity into the datasets, complicating the prediction task for the machine learning models. The Augmented Dickey-Fuller (ADF) test confirmed the initial non-stationarity of the USA, the EU27&UK, and India datasets, which required first-order differencing to achieve stationarity. By stabilising the time series through differencing, the models were able to better handle these seasonal effects, leading to improved predictive performance. Moreover, ML models often benefit from feature engineering (Fan et al. 2019; Wang et al. 2022). While differencing may sometimes result in the loss of valuable information, it also creates new complex features and patterns that ML models can capture, thereby enhancing the model's performance.

**Fig. 8:**
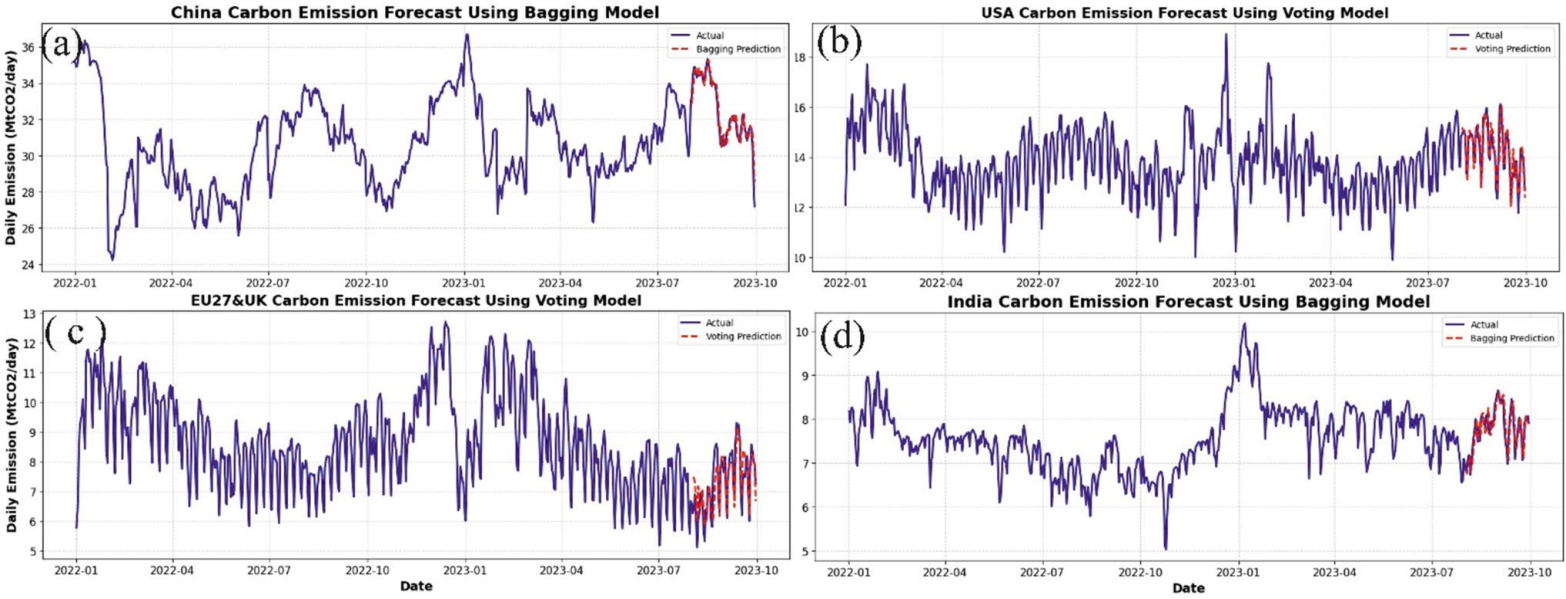
Plots of actual and predicted emissions for (**a**) China, (**b**) USA, (**c**) EU27&UK, and (**d**) India using the best ML models

Additionally, the model’s performance improved when ensemble techniques such as bagging and voting were applied, increasing the *R*^2^ value from a range of 0.677–0.906 to 0.720–0.926 across the four regions. Overall, the ML model performed well in predicting daily emissions, as demonstrated by the fitting curve of the best ML model across each region, as illustrated in Fig. 8. The best-performing ML models are the bagging model (China/India dataset) and the voting model (USA/EU27&UK dataset). These results highlight the importance of handling seasonality and non-stationarity in time series forecasting to improve the accuracy of CO_2_ emission predictions.

#### Deep learning models

**Fig. 9:**
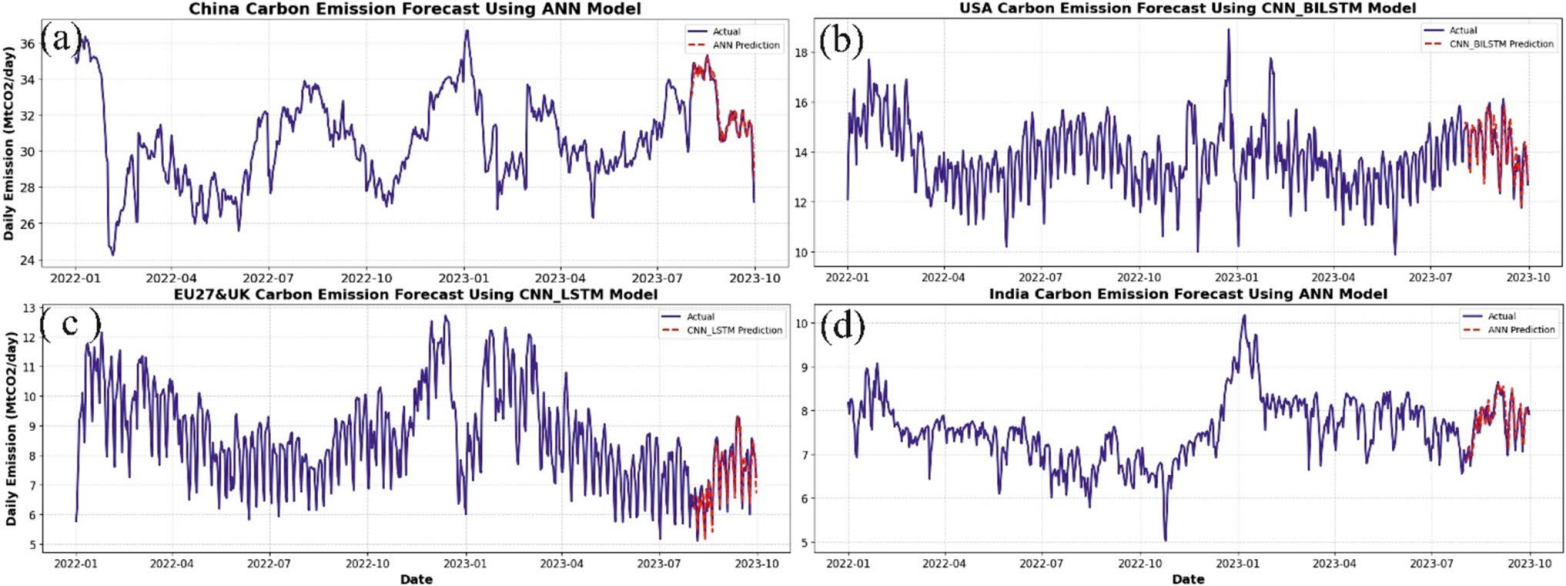
Plots of actual and predicted emissions for (**a**) China, (**b**) USA, (**c**) EU27&UK, and (**d**) India using the best DL models

Since differencing improves the performance of ML models, DL models (ANN LSTM, BILSTM, GRU, and hybrid models) were also applied to the differenced dataset. Notably, the hybrid combination of CNN-RNN slightly improved the performance of the RNN models for all regions except for the Indian dataset, where the performance remained relatively unchanged. As discussed in the “Dataset” section, India’s dataset exhibits the least variability and seasonality compared with other regions, as indicated by its lower standard deviation. This reduced variability limits the complexity that DL models, such as CNN-RNN hybrids, can leverage to capture intricate patterns. Additionally, while first-order differencing successfully addresses non-stationarity, the lack of rich seasonal and nonlinear patterns in India’s data may have restricted the models from achieving further performance improvements. As detailed in Table 9, the *R*^2^ values of the CNN-GRU model improved from 0.905 to 0.923, 0.755 to 0.803, and 0.841 to 0.878 for China, the USA, and the EU27&UK, respectively. According to Duan et al. (2022), this blend enables the model to acquire temporal and spatial characteristics from the data, enhancing overall performance. Overall, the predictive accuracy of the DL model is closely aligned with the actual data, as shown in Fig. 9. The best-performing DL models were ANN (for the China and India datasets), CNN-BILSTM (for the USA dataset), and CNN-LSTM (for the EU27&UK datasets).

**Table 9:**
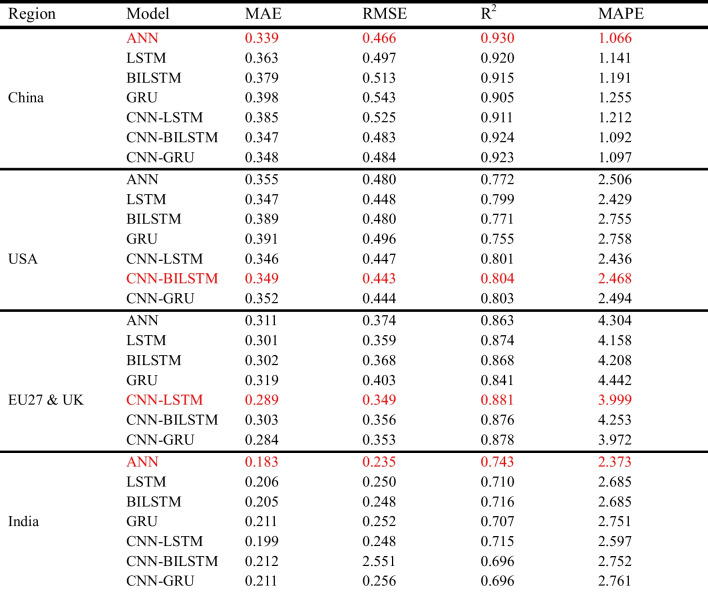
Machine learning models performance evaluation

*The red mark indicates the best models*

## Discussion

### Comparative analysis of the performance of the best statistical, ML, and DL models

**Fig. 10:**
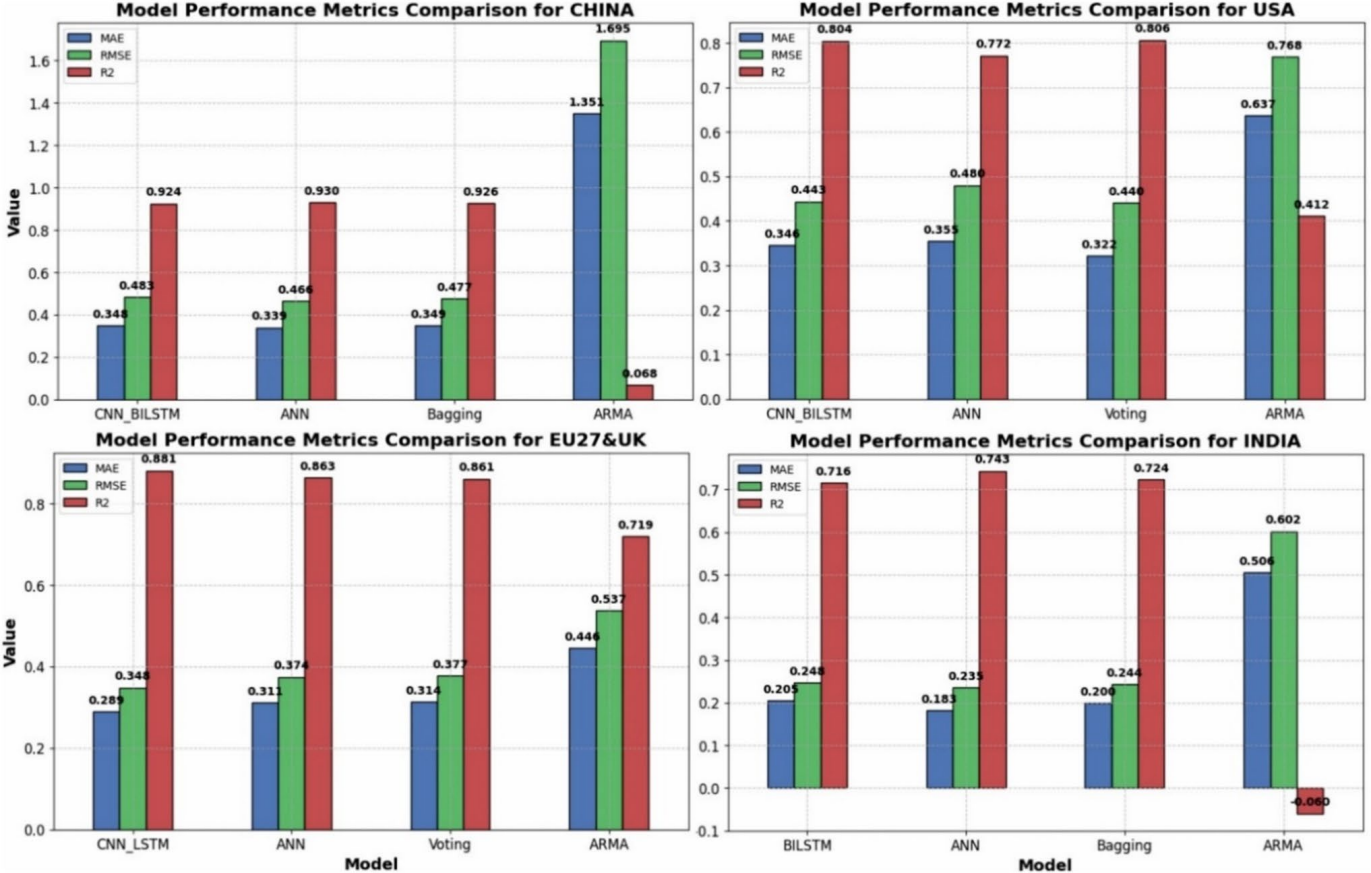
Comparative assessment of the best statistical, ML, and DL models across the four regions

Figure 10 compares the best statistical, ML, and DL models across the four regions. The results show that both the ML and DL models consistently outperform traditional statistical models in predicting daily CO_2_ emissions, with lower MAE and RMSE values and higher *R*^2^ scores. Statistical models, with *R*^2^ values ranging from − 0.060 to 0.719 and RMSE values between 1.695 and 0.537, struggled to capture the complex, nonlinear patterns present in the emissions data. In contrast, the ML and DL models achieved *R*^2^ values from 0.714 to 0.932 and RMSE values as low as 0.247, indicating more accurate and robust predictions. These findings align with the research of Li and Zhang (2023), who reported that ML and DL models are better equipped to model nonlinear datasets, as is the case with daily CO_2_ emissions. This ability to handle nonlinearities likely explains the superior performance of the ML and DL models in our study.

The statistical models struggled to capture the high variability and nonlinearity inherent in the emission datasets. These models rely on assumptions of stationarity, which are often violated in real-world CO_2_ emissions data due to fluctuating economic activities, weather conditions, and policy changes. The inability of statistical models to adequately handle these dynamic and nonlinear factors explains their lower performance in this study. Future work could explore hybrid approaches that combine statistical models with ML/DL techniques to manage these complexities better, potentially using statistical models for short-term forecasts and ML/DL models for long-term predictions. Such an approach would leverage the strengths of both methods, offering more flexibility in handling different data characteristics.

Model performance varies across regions, suggesting that the specific characteristics of each dataset influence the best-performing model. For example, the ANN model performed best for the China and India datasets, while the ML-voting model performed excellently for the USA dataset, and the CNN-LSTM model achieved the highest accuracy for the EU27&UK dataset. These variations can be attributed to differences in regional data characteristics, such as the degree of nonlinearity, variability, and seasonality. As noted by Oreski et al. (2017), model performance is highly dependent on the data attributes, reinforcing the need for region-specific modelling approaches.

Furthermore, the performances of the ML and DL models were relatively similar. For example, metrics for CNN-BILSTM (RMSE = 0.483, *R*^2^ = 0.924), ANN (RMSE = 0.466, *R*^2^ = 0.930), and ML-bagging (RMSE = 0.477, *R*^2^ = 0.926) were reported for the China dataset. This similarity may be attributed to the relatively small sample size (638 data points) used in this study. When trained on larger datasets, deep learning models typically show a clear advantage over ML models, as they can learn more complex patterns over time. Romeiko et al. (2020) reported that the performance gap between ML and DL models widens with increasing dataset size, suggesting that DL models would likely outperform ML models with larger datasets.

**Fig. 11:**
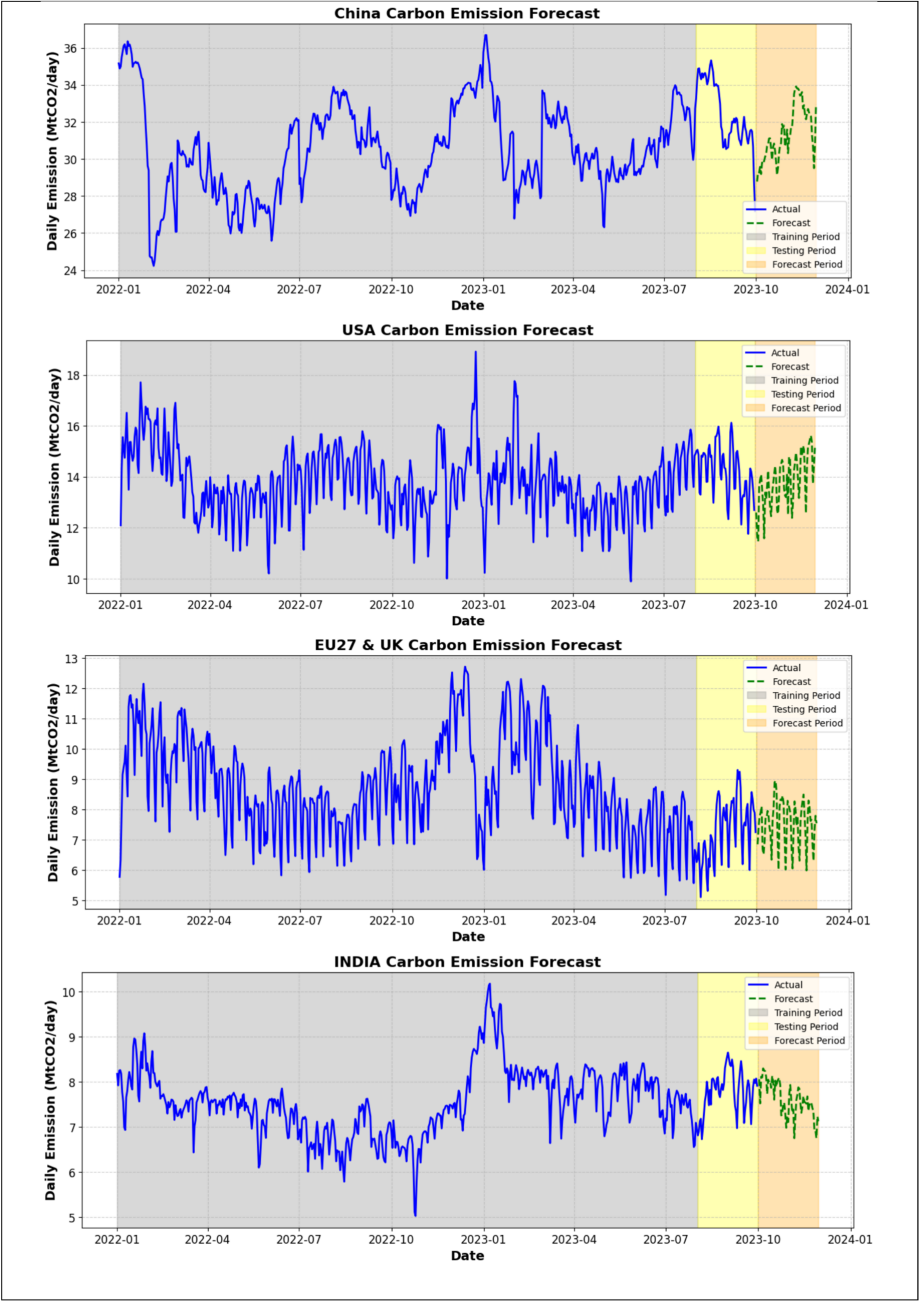
CO_2_ emissions forecast for the next 60 days across the four regions

The computational complexity of deep learning (DL) models, which often require specialised hardware such as GPUs or TPUs, may limit their practical application in environments with limited resources. Following the principle of **Occam’s Razor**, which suggests favouring simpler solutions when they achieve comparable results, machine learning (ML) models provide a compelling alternative. ML models, particularly ensemble techniques such as voting and bagging, offer a more computationally efficient approach while maintaining high predictive accuracy. This balance between performance and computational demand makes ML models more suitable for real-world applications where data or hardware resources are limited. As shown in Fig. 11, the ML models were used to forecast daily emissions for the next 60 days across the four regions. The forecast indicates a similar trend to that of the previous winter period, with a gradual increase in CO_2_ emissions as regions progress deeper into winter. This increase in emissions is attributed to the heightened demand for heating, which leads to greater coal consumption and elevated CO_2_ emissions. These insights are crucial for policymakers in anticipating seasonal spikes in emissions and implementing appropriate mitigation strategies.

#### Comparison with previous studies

Compared with our study, Li and Zhang (2023) reported an *R*^2^ of 0.96 when LSTM was used to forecast daily emissions from China during the COVID-19 period, requiring 3000 epochs for training. In contrast, our study achieved an *R*^2^ of 0.93 with only 200 epochs, demonstrating the computational efficiency of our approach. Additionally, our use of differencing significantly improved model accuracy, a factor not considered in previous studies. This enhancement reduces computational demand while maintaining high predictive accuracy, making it suitable for real-time applications in policymaking.

Other studies have also applied machine learning and deep learning techniques to emission prediction, often with a narrower geographic focus or different timeframes. For example, Huang et al. (2019) focused on carbon emissions in China, whereas Song et al. (2023) examined emissions in South Korea. These studies used models on an annual or regional scale. In contrast, our study provides a broader and more dynamic assessment of daily emissions data across four major polluting regions: China, India, the USA, and the EU27&UK. This geographic breadth allows for a more comprehensive analysis, enabling policymakers to set both short- and long-term emission reduction targets.

Furthermore, the robust performance of our models on smaller datasets makes them suitable for less developed countries where data collection and availability are limited, allowing these regions to better participate in global climate change mitigation efforts. This scalability and adaptability set our study apart from others that focused on larger, more comprehensive datasets. Finally, unlike previous studies that focused predominantly on annual predictions, our use of daily emissions data provides timely insights into emissions trends. This real-time capability is essential for governments aiming to make rapid policy adjustments, particularly during periods of economic recovery or energy transition, such as those faced by the major polluting regions analysed in this study.

## Conclusion

The accurate prediction of daily real-time CO_2_ emissions holds significant importance for governmental initiatives to mitigate global warming. This study conducted a comparative analysis of the performance of four statistical, three ML, and seven DL models in predicting daily CO_2_ emissions across four regions. The models were evaluated using four evaluation metrics, and the following conclusions were drawn from this study.

- The ML and DL models, with higher *R*^2^ (0.714–0.932) and lower RMSE (0.480–0.247) values, respectively, outperformed the statistical model, which had *R*^2^ (− 0.060–0.719) and RMSE (1.695–0.537) values, in predicting daily CO_2_ emissions across all four regions.
- The performance of the ML models significantly improved through a combination of differencing and the application of ensemble techniques (voting and bagging), resulting in an average increase of 9.6% in *R*^2^, expanding the range from 0.677–0.906 to 0.720–0.926 across all four regions.
- The performance of the RNN models was enhanced by a hybrid combination of CNN-RNN, which improved the *R*^2^ values across all the regions and increased the range from 0.710–0.923 to 0.743–0.930.

Overall, the performances of both the ML and DL methods were relatively similar. However, owing to the high computational requirements associated with DL models, the recommended models for daily CO_2_ emission prediction are ML models that use the ensemble technique of voting and bagging. These models are then employed to forecast the emission trends across the four regions for the next 60 days. This study contributes to the expanding body of research on carbon emission prediction, offering valuable insights into the effectiveness of short-term CO_2_ emission forecasts. These findings have significant implications for policymakers, enabling more dynamic adjustments to emission reduction strategies based on daily data and ultimately supporting more effective global warming mitigation efforts.

### Policy implications

Using machine learning and deep learning models to predict daily CO_2_ emissions can enable timely policy adjustments and help prevent emission spikes, especially during economic recovery phases. As regions such as China, India, the USA, and the EU strive to meet carbon reduction targets, accurate daily emissions data will equip governments with tools to set short-term targets and make real-time interventions, allowing them to adjust policies more frequently than annual predictions allow.

For example, as economies recover post-COVID-19, monitoring real-time emissions data can prevent emissions from rebounding to pre-crisis levels, ensuring progress toward carbon neutrality. Governments should use these data to implement targeted policies in high-emission sectors such as energy, transportation, and industry:

- **Energy sector:** Policies that encourage the expansion of renewable energy sources (such as solar, wind, and biomass) are vital. Promoting the use of clean energy over fossil fuels will help lower emissions in energy-intensive countries such as China and the USA.
- **Transportation sector**: Investment in sustainable transport infrastructure, the widespread adoption of electric vehicles, and green public transportation systems significantly contribute to reducing emissions from ground transportation, a major source of CO_2_ in regions such as the EU and the USA.
- **Industrial sector**: Adopting clean technology and energy-efficient practices in manufacturing and heavy industries can help curb emissions in countries with high industrial output, such as China and India.

Additionally, promoting low-carbon lifestyles and raising public awareness of energy conservation will further support emission reductions. This can be achieved through educational campaigns, financial incentives for energy-saving practices, and the integration of sustainability principles into everyday life. Governments must actively encourage the adoption of energy-efficient practices across all sectors by implementing supportive regulations and offering incentives for innovation. These efforts will align policies with sustainable development goals, ultimately contributing to a greener, more resilient future.

### Limitations and future work

Despite the advancements in daily CO_2_ emissions prediction demonstrated in this study, reliance on univariate data is a key limitation. Important exogenous factors were not considered, such as daily renewable energy consumption, population data, GDP, and daily pump prices, which significantly influence CO_2_ emissions. The exclusion of these factors may result in less comprehensive predictions. A major challenge is that many of these variables are only available as monthly or yearly data, making them difficult to incorporate into daily models.

Our future research will address this limitation by employing a multivariate approach, as Li and Zhang (2023) suggested, which would allow for incorporating these exogenous factors, thus potentially enhancing the models’ accuracy and robustness. This approach is particularly effective when hybrid machine learning and deep learning techniques are utilised, as Juliet et al. (2024) demonstrated. Furthermore, developing methods to integrate these diverse data types could result in a more precise and comprehensive model for predicting emissions. In addition, future work could explore hybrid approaches that combine statistical models with ML/DL techniques to better manage these complexities, potentially using statistical models for short-term forecasts and ML/DL models for long-term predictions. Such an approach would leverage the strengths of both methods, offering more flexibility in handling different data characteristics.

Additionally, while this study focuses on the overall CO_2_ emissions from China, India, the USA, and the EU27&UK, future work will aim to explore the sectoral contributions to daily emissions across these regions. Specifically, we will investigate how different sectors (aviation, ground transport, power, industrial, and residential) contribute to emission patterns and how these contributions vary across regions. Understanding these sectoral contributions will provide detailed insights that are essential for developing region-specific emission reduction strategies.

Moreover, the scope of our future studies will expand to a more global or continental level, exploring daily CO_2_ emission trends on a broader scale. This would lay the groundwork for incorporating short-term CO_2_ mitigation policies globally, providing critical insights into emission trends across different regions and facilitating the creation of more effective global policies. In summary, while ML and deep learning models have shown promise, future research should move beyond the limitations of univariate models and focus on multivariate approaches to improve accuracy and policy relevance.

## Data Availability

The dataset used for this study is available from the Carbon Monitor project repository (https://carbonmonitor.org) and is made freely available to the public under a fair use open data policy.

## Appendix

Table 10

**Table 10:** Nomenclature list

Symbol	Nomenclature
CO_2_	Carbon Dioxide
MtCO_2_/day	Million Tons of CO_2_ per Day
ML	Machine Learning
DL	Deep Learning
ARIMA	Autoregressive Integrated moving average
SARIMA	Seasonal Autoregressive Integrated Moving Average
ARMA	Autoregressive Moving Average
ANN	Artificial Neural Network
SVM	Support Vector Machine
RF	Random Forest
GB	Gradient Boosting
LSTM	Long Short-Term Memory
GRU	Gated Recurrent Unit
BILSTM	Bidirectional Long Short-Term Memory
R^2^	Coefficient of Determination
RMSE	Root Mean Square Error
MAE	Mean Absolute Error
MAPE	Mean Absolute Percentage Error
ADF	Augmented Dickey-Fuller Test
PACF	Partial Autocorrelation Function
ACF	Autocorrelation Function
${f}_{t}$	Forget Gate
${i}_{t}$	Input Gate
${O}_{t}$	Output Gate
${r}_{t}$	Reset Gate
${z}_{t}$	Update Gate
${C}_{t}$	Memory Cell

## Code availability

The code used for the analysis is available upon request to enable full reproducibility of the model implementation and results.
